# Evidence‐based management strategies for endocrine complications after pituitary adenoma surgery

**DOI:** 10.1002/ibra.12156

**Published:** 2024-05-23

**Authors:** Xiaoju Miao, Zhongmin Fu, Xian Luo, Jun Wang, Zhangzhu Ren, Yuanyuan Wang, Guoping Mei, Shunwu Xiao

**Affiliations:** ^1^ The first ward of the Neurosurgery Department Affiliated Hospital of Zunyi Medical University Zunyi China

**Keywords:** complication management, endocrine, evidence‐based medicine, pituitary adenoma

## Abstract

This study aims to provide a clinical reference for the management of endocrine complications in pituitary tumor patients by synthesizing recent evidence for domestic and international management strategies. Based on the PIPOST (Population, Intervention, Professional, Outcome, Setting, and Type of Evidence) framework, evidence‐based medicine targets were determined. Electronic decision support systems, guideline websites, and databases were searched to identify the best evidence on postoperative endocrine complications in pituitary tumors. The PICO (Patient, Intervention, Comparison, Outcome) principle was used to construct the search strategy, and the studies from the past 10 years (July 2013 to July 2023) were included. A total of 11 articles were included, including four guidelines, two expert consensus statements, one systematic review, one best practice article, and three randomized controlled trials. We obtained evidence on five aspects (endocrine assessment, secondary adrenal insufficiency management, water metabolism disorder management, special population management, and follow‐up management) with a total of 30 pieces of evidence. Clinical healthcare professionals should focus on the care and follow‐up of patients with postoperative complications, such as adrenal insufficiency, temporary or permanent diabetes insipidus, and hyponatremia. Future research should involve large sample sizes, long‐term follow‐ups, and multicenter studies to further clarify the protocols for fluid restriction, diet, and hormone use.

## Graphical abstract

Based on the PIPOST (Population, Intervention, Professional, Outcome, Setting, and Type of Evidence) framework, evidence‐based medicine questions were developed, and a top‐down search was conducted according to the 6S model of the evidence pyramid. The literature sources were best practices, guidelines, expert consensuses, systematic reviews, meta‐analyses, or randomized controlled trials. AGREE II (Appraisal of Guidelines for Research and Evaluation II), AMSTAR 2 (Assessment of Multiple Systematic Reviews 2), ROB2 (Risk of Bias 2.0), and JBI (Joanna Briggs Institute) Expert Opinion Assessment Tool were used for literature quality assessment. Evidence was synthesized on five aspects: endocrine assessment, secondary adrenal insufficiency management, water metabolism management, special populations management, and follow‐up management.

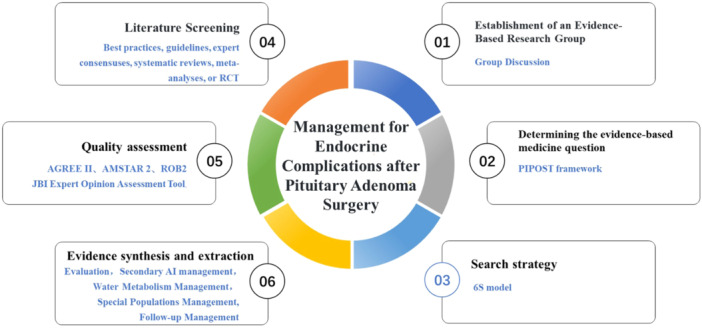

## 1 INTRODUCTION

Pituitary adenoma is a benign tumor that originates from the pituitary gland and accounts for approximately 10%–15% of intracranial tumors.^1^ Surgery is the primary treatment for adrenocorticotropic hormone‐secreting tumors, growth hormone (GH)‐secreting tumors, visual impairment due to optic nerve or chiasm compression, and pituitary apoplexy accompanied by visual field defects.^2^ However, endocrine complications often occur after pituitary tumor surgery, which account for approximately 11% of all postoperative complications. A retrospective analysis in the United States showed that among 1153 consecutive transsphenoidal pituitary adenoma resections performed between 1992 and 2017, endocrine complications included transient diabetes insipidus (DI; 4.3%), permanent DI (0.3%), symptomatic hyponatremia (4.2%), hormone deficiencies affecting any axis (3.6%), and adrenal insufficiency (AI, 0.2%).^3^ Another report from Virginia (1995–2001) reported a higher incidence of DI, which reached 18.3% immediately after surgery, with only 2% of patients having permanent DI.^4^ It has been shown that hyponatremia generally peaks approximately 5–7 days after discharge in patients with pituitary adenoma.^5^, ^6^ In addition, delayed symptomatic hyponatremia (DSH) is a common reason for readmission within 30 days after surgery, accounting for 25%–50% of readmissions after pituitary surgery.^7^, ^8^, ^9^ Symptoms of DSH include nausea, vomiting, and more severe symptoms such as seizures, altered mental status, and even death.^10^, ^11^ In response to these challenges, some institutions advocate for postoperative electrolyte screening for all patients undergoing pituitary adenoma surgery to facilitate early detection and intervention for DSH. These protocols include electrolyte supplementation, perioperative steroid use, frequent monitoring of postoperative serum sodium levels after discharge, and fluid restriction.^6^, ^12^, ^13^

Due to differences in protocols among different centers, a unified consensus has not been reached yet. Given the high frequency of endocrine complications in patients after pituitary adenoma surgery and the lack of guidance for managing these complications, the purpose of this study was to summarize existing strategies for managing endocrine complications postpituitary adenoma surgery using an evidence‐based medicine approach. This study will provide guidance for the management of endocrine complications among patients undergoing pituitary adenoma surgery.

## 2 METHODS

### 2.1 Determining the evidence‐based medicine targets

The present study was registered at the Evidence‐Based Medicine Center of Fudan University (http://ebn.nursing.fudan.edu.cn/, registration number ES20233124). The framework containing PIPOST (Population, Intervention, Professional, Outcome, Setting, and Type of Evidence) was utilized to determine the evidence‐based targets. The targets were finally decided as follows. Population (P): Patients undergoing pituitary adenoma surgery. Intervention (I): Management strategies for postoperative complications in patients with pituitary adenoma. Professionals (P): Neurosurgeons, nurses, and patient family members. Outcome measures (O): Incidence of DI, DSH, hypernatremia, AI, any postoperative hormone deficiency, average daily urine output, length of stay, and required amounts of antidiuretic hormone (ADH) and glucocorticoid. Setting (S): Neurosurgery ward and home. Types of evidence sources (T): Clinical guidelines, evidence‐based guidelines, expert consensuses, systematic reviews, best practices, and randomized controlled trials (RCTs). According to such targets, our study aims to address the clinical management of postoperative endocrine complications in patients with pituitary adenomas.

### 2.2 Search strategy

The PICO (Patient, Intervention, Comparison, Outcome) principle was used to construct the search query with the following keywords: “pituitary tumor,” “transsphenoidal surgery,” and “endocrine complication.”

We searched computerized decision support systems for relevant information, such as BMJ Best Practice and UpToDate. We also searched guideline websites, including the Agency for Health Care Research and Quality, Guidelines International Network, National Institute for Health and Care Excellence, Scottish Intercollegiate Guidelines Network, Registered Nurses' Association of Ontario, and JBI (Joanna Briggs Institute). Additionally, relevant association websites were searched, such as the International Pituitary Association, Association of the Scientific Medical Societies in Germany, American Medical Association, American Cancer Research Society, and European Society of Endocrinology. Finally, the following databases were searched, including ClinicalTrials.gov, PubMed, Embase, OvidSP, CINAHL, Cochrane Library, China National Knowledge Infrastructure, WANFANG Database, and Chinese Clinical Guidelines (YiMaiTong). The search was performed from July 2013 to July 2023, and there were no language restrictions.

### 2.3 Inclusion and exclusion criteria

**Figure 1:**
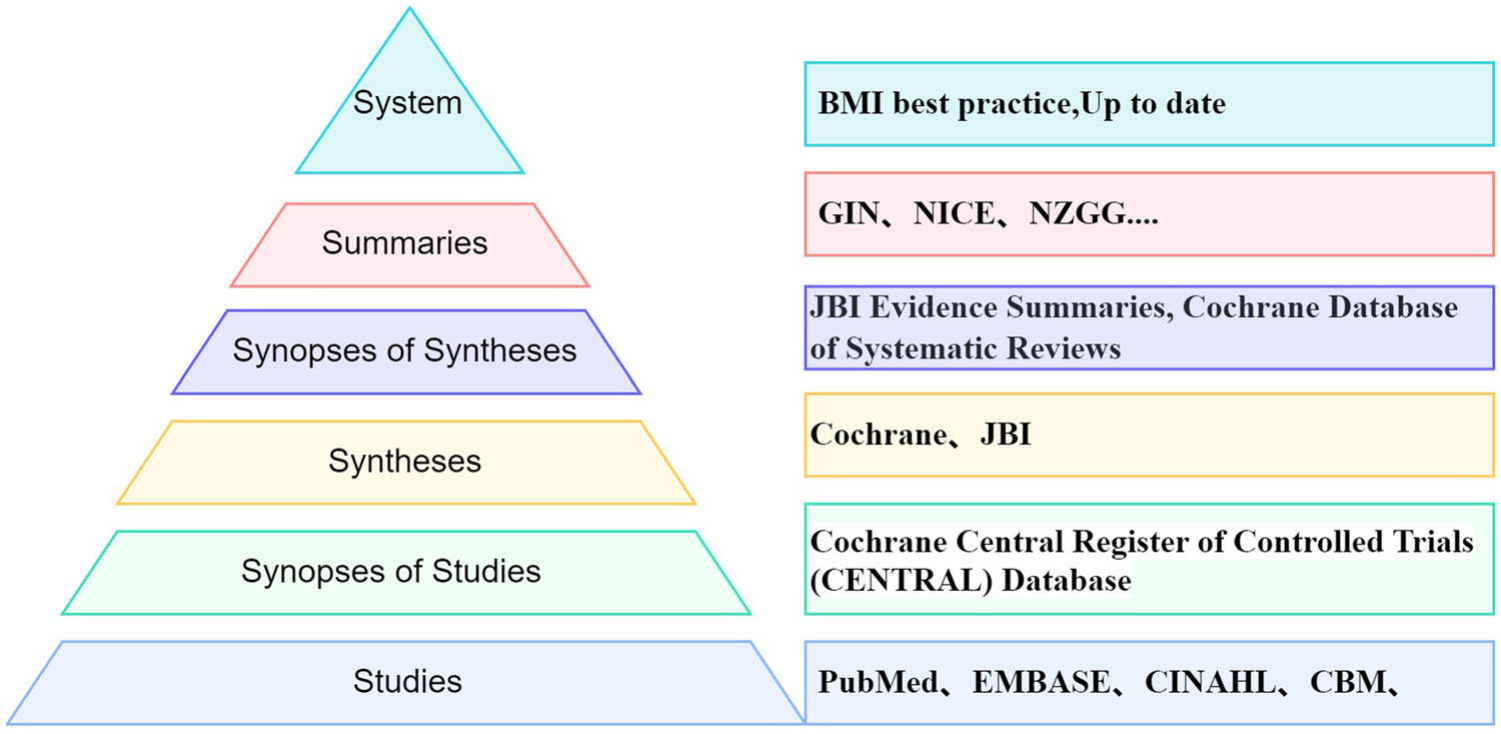
Evidence‐based medicine 6S model. Each “S” in the pyramid represents a specific type of resource, arranged from top to bottom as follows: Systems, Summaries, Synopses of Syntheses, Syntheses, Synopses of Studies, and Studies. GIN, Guidelines International Network; NICE, National Institute for Health and Care Excellence; JBI, Joanna Briggs Institute. [Color figure can be viewed at wileyonlinelibrary.com]

The inclusion criteria were as follows: (1) the studies focusing on postoperative patients with pituitary adenoma, specifically addressing the evaluation, management, and prevention of postoperative endocrine complications and (2) the study types including best practices, guidelines, expert consensuses, expert opinions, systematic reviews, meta‐analyses, or RCTs. According to the 6S model (Figure 1), a framework used to categorize and evaluate different types of evidence, clinical evidence can be retrieved from the top to the bottom of the pyramid in turn, starting from Systems to Study, which indicates that the primary study has the lowest level of quality evidence in this model. Given this hierarchy, RCTs, with higher quality than the primary study, were prioritized and included exclusively in the primary study to ensure the synthesis of the best quality evidence, while cohort studies, retrospective studies, and other types of studies were not subjected to evidence synthesis.

The exclusion criteria were as follows: (1) reviews lacking systematic search and methodological evaluation, animal studies, basic science papers, case–control studies, translated versions of the studies, administrative database studies, or case reports and (2) studies that did not report prevention or treatment strategies for pituitary adenoma‐related endocrine complications or were assessed as low quality.

### 2.4 Quality assessment

Two researchers who had received training in evidence‐based practice independently conducted the literature screening and data extraction. In the event of disagreements, a third expert in evidence‐based medicine participated in collaborative discussions to reach a consensus.

AGREE II (Appraisal of Guidelines for Research and Evaluation II)^14^ was used to assess the quality of the guidelines. The tool consists of six domains and 23 items, with each item scored on a scale of 1–7. The score and standardized percentage are calculated for each domain using the formula: (actual score − theoretical minimum score)/(theoretical maximum score − theoretical minimum score). Based on the standardized percentage, the guidelines are classified into three levels: level A recommendation is given when the standardized percentage is ≥60% in all domains; level B recommendation is given when there are ≥3 domains with a standardized percentage ≥30%, but some domains have less than 60%; level C recommendation is given when there are ≥3 domains with a standardized percentage <30%, indicating no recommendation.

The quality assessment of the expert consensus was conducted using the JBI Expert Opinion Assessment Tool. This tool consists of six assessment items: “Whether the sources of the opinions are clearly stated,” “Whether the opinions are from influential experts in the field,” “Whether the opinions presented are centered on the interests of the relevant population,” “Whether the conclusions are based on the results of analysis, and whether the expression of opinions is logical,” “Whether other existing literature has been referenced,” and “Whether the opinions presented are inconsistent with previous literature.” Researchers can provide judgments of “yes,” “no,” “unclear,” or “not applicable” when evaluating. When all items are rated as “yes,” it indicates a high quality of expert consensus.

The quality assessment of the systematic reviews was conducted using the AMSTAR 2 (Assessment of Multiple Systematic Reviews 2) tool.^15^ The tool consists of a total of 16 items, with items 2, 4, 7, 9, 11, 13, and 15 being critical items and the rest being noncritical items. Each item evaluation result is categorized as “Yes (Y),” “Partly Yes (PY),” or “No (N).” When the evaluation result is “No,” it is considered not met. If 0 or 1 noncritical item is not met, it is rated as “high quality.” If more than one noncritical item is not met, it is rated as “moderate quality.” If one critical item is not met with or without noncritical items not being met, it is rated as “low quality.” If more than one critical item is not met with or without noncritical items not being met, it is rated as “very low quality.” Two researchers independently assessed the quality of RCTs using the ROB2 (Risk of Bias 2.0) tool,^16^ which was published by the Cochrane Collaboration in 2019. Six domains of bias were evaluated: bias in the randomization process, bias due to deviations from intended interventions, bias in the measurement of outcomes, bias due to missing outcome data, bias in the selection of reported results, and overall bias.

### 2.5 Evidence synthesis and extraction

We use the Australian JBI evidence pregrading and evidence recommendation level system to grade the included evidence. Depending on the type of study design, the evidence is classified into levels 1–5 (with level 1 being the highest and level 5 being the lowest). Based on the effectiveness, feasibility, appropriateness, and clinical significance of the evidence, combined with the JBI principles of recommendation strength, the recommendation levels are divided into grade A recommendation and grade B recommendation. The research team independently extracted and synthesized the relevant evidence from the included studies. The evidence was crosschecked and integrated by the evidence synthesis team, and the evidence themes and recommendation levels were determined through expert meetings (with recommendation level A being more favorable than level B). During evidence synthesis, consistent findings were amalgamated, prioritizing high‐quality and recent authoritative studies in cases of inconsistent evidence. If there was only one source of evidence, the evidence from that source was adopted.

## 3 RESULTS

### 3.1 General characteristics of the included studies

**Figure 2:**
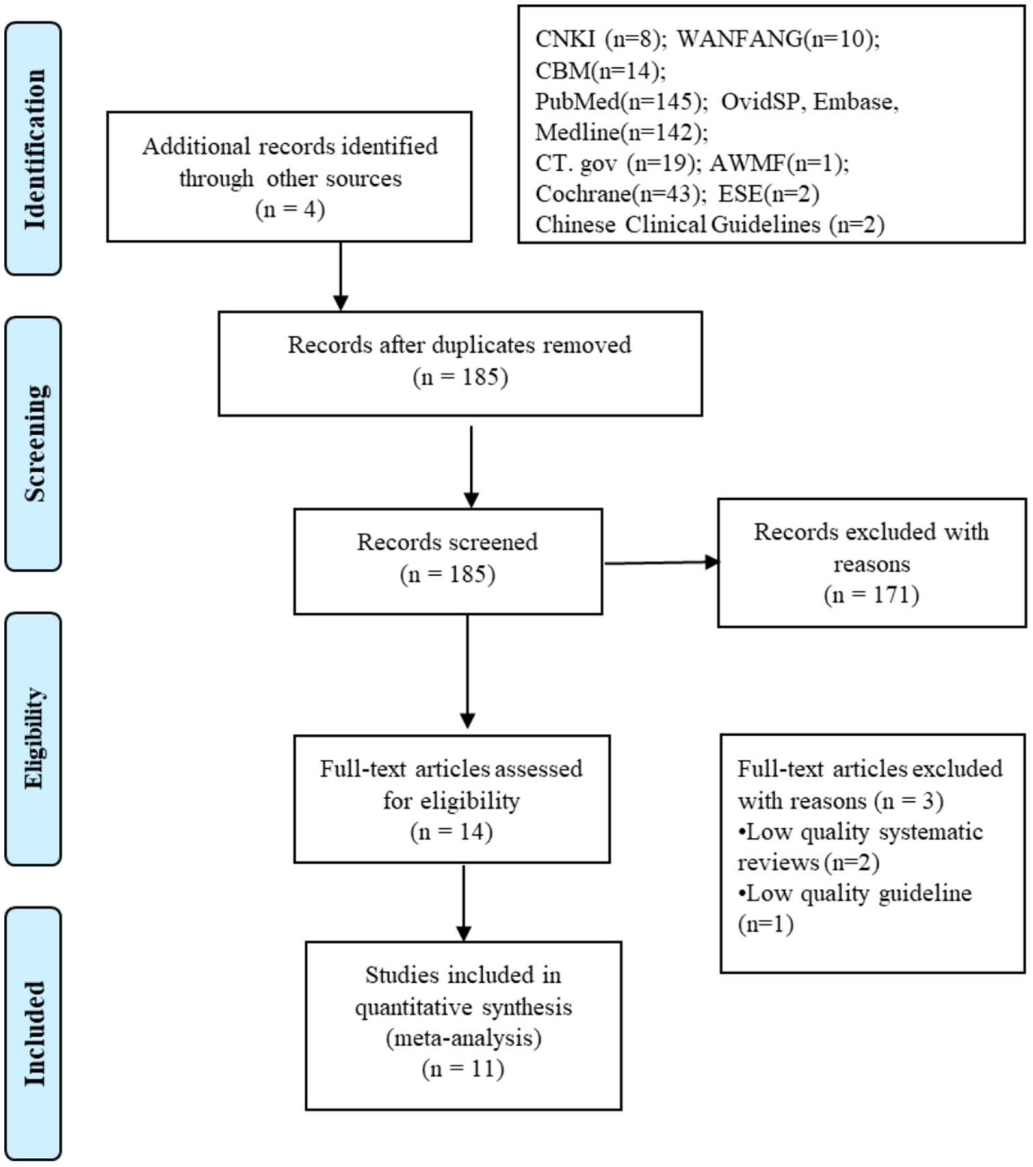
Literature screening process flowchart. AWMF, Association of the Scientific Medical Societies in Germany; CNKI, China National Knowledge Infrastructure; CT.gov, ClinicalTrials.gov; ESE, European Society for Endocrinology.

Our search initially yielded 390 potentially relevant studies, which were reduced to 185 studies after removing duplicates. Following title and abstract screening, 14 relevant studies were identified for full‐text selection. Upon reading the full text, two systematic reviews and one guideline were excluded due to inadequate quality, resulting in the final inclusion of 11 studies (Figure 2). The included studies comprised four guidelines,^17^, ^18^, ^19^, ^20^ two expert consensus statements,^21^, ^22^ one systematic review,^23^ one best practice,^24^ and three RCTs.^25^, ^26^, ^27^ The basic characteristics of the included articles are presented in Table 1.

**Table 1:** General characteristics of included studies.

Included studies	Publication year	Source of literature	Type of literature	Evidence topic
Lamas et al.^19^	2014	PubMed	Clinical guideline	Management of diabetes insipidus and syndrome of inappropriate antidiuretic hormone secretion after pituitary surgery
Nieman et al.^20^	2015	ESE	Clinical guideline	Treatment of Cushing's syndrome
Fleseriu et al.^18^	2016	ESE	Clinical guideline	Hormonal replacement in hypopituitarism in adults
Deutschbein et al.^17^	2021	AWMF	Clinical guideline	Clinically Nonfunctioning pituitary tumors
Prete et al.^24^	2017	PubMed	Best practice	Management of patients after pituitary surgery
Chinese Pituitary Adenoma Collaboration Group^22^	2015	Chinese Medical Association	Consensus	Surgical treatment of pituitary adenomas in China
Tritos et al.^21^	2022	OvidSP	Consensus	Endocrine management of patients undergoing transsphenoidal surgery for pituitary adenomas
Yu et al.^23^	2022	PubMed	Systematic review	Delayed symptomatic hyponatremia in transsphenoidal surgery
Sterl et al.^27^	2019	PubMed	RCT	Withholding perioperative steroids in patients undergoing transsphenoidal resection for pituitary disease
Koundal et al.^26^	2021	PubMed	RCT	Dietary diabetes insipidus bundle on the severity of postoperative fluid imbalance in pituitary region tumors
Guo et al.^25^	2022	PubMed	RCT	Safety of withholding perioperative hydrocortisone for patients with pituitary adenomas

Abbreviations: AWMF, Association of the Scientific Medical Societies in Germany; ESE, European Society for Endocrinology; RCT, randomized controlled trial.

### 3.2 Results of the quality evaluation of the included studies

**Figure 3:**
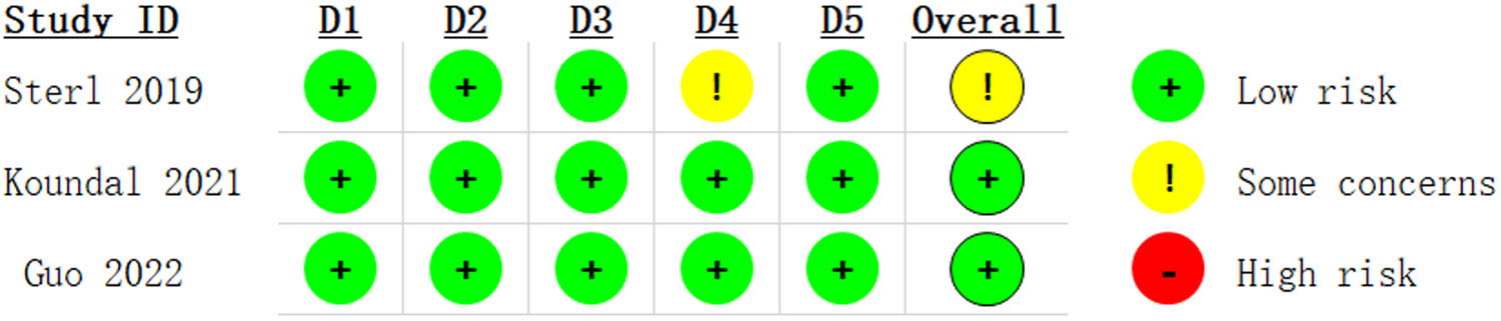
Results of quality assessment for randomized controlled trials (RCTs). Using the Risk of bias 2.0 tool to assess the quality of RCT, D1 represents bias arising from the randomization process, D2 represents bias due to deviations from intended interventions, D3 represents bias due to missing outcome data, D4 represents bias in the measurement of the outcome, and D5 represents bias in the selection of the reported result. [Color figure can be viewed at wileyonlinelibrary.com]

This study included four guidelines and one clinical practice. The specific evaluation results are detailed in Table 2. Two expert consensuses were included, and all evaluation items in these consensuses were rated as “Yes,” showing high quality. One high‐quality systematic review was included, and all evaluation items except Item 8 (“Did the authors of the systematic review describe the included studies in detail?”) were rated as “Yes.” Among the three RCTs, only the study by Sterl et al.^27^ was rated as “some concerns,” while the remaining two RCTs were considered low‐risk studies. The evaluation results are presented in detail in Table 3 and Figure 3.

**Table 2:** Distribution of AGREE II score results.

Guidelines	Year	Scope and objective (%)	Participants (%)	Rigor (%)	Clarity (%)	Practicality (%)	Independence (%)	Number of domains with ≥60%	Number of domains with <30%	Recommendation	Recommendation level
Lamas et al.^19^	2014	80.6	33.3	29.2	69.4	39.6	75	3	1	Yes	B
Nieman et al.^20^	2015	91.7	66.7	72.2	94.4	60.4	95.8	6	0	Yes	A
Fleseriu et al.^18^	2016	94.4	86.1	65.3	91.7	45.8	95.8	5	0	Yes	B
Prete et al.^24^	2017	66.7	35.6	23.6	66.7	29.2	75	3	2	Yes	B
Deutschbein et al.^17^	2021	88.9	72.2	65.3	86.1	43.8	83.3	5	0	Yes	B

Abbreviation: AGREE II, Appraisal of Guidelines for Research and Evaluation II.

**Table 3:** Results of Risk of Bias 2.

Study (year)	Bias arising from the randomization process	Bias due to deviations from intended interventions	Bias due to missing outcome data	Bias in measurement of the outcome	Bias in selection of the reported result	Overall bias
Sterl et al. (2019)^27^	Low risk	Low risk	Low risk	Some concerns	Low risk	Some concerns
Koundal et al. (2021)^26^	Low risk	Low risk	Low risk	Low risk	Low risk	Low risk
Guo et al. (2022)^25^	Low risk	Low risk	Low risk	Low risk	Low risk	Low risk

### 3.3 Summary and description of the evidence

In this study, the evidence from the 11 included articles was classified, summarized, and evaluated, resulting in five aspects: endocrine assessment, management of AI, management of water metabolism disorders, management of special populations, and follow‐up management. A total of 30 pieces of evidence were obtained, including 10 pieces of evidence with A‐level recommendations and 20 pieces of evidence with B‐level recommendations. High‐quality evidence suggests that “pituitary adenoma patients with normal hypothalamic‒pituitary‒adrenal (HPA) axis function should not use glucocorticoids postoperatively,” “patients with secondary adrenal insufficiency receive glucocorticoid replacement therapy after surgery,” “to screen for DI and syndrome of inappropriate antidiuretic hormone (SIADH), it is necessary to measure urine volume and fluid intake, measure urine specific gravity daily, and measure serum sodium every 6–12 h until 7–10 days postoperatively,” “patients are advised to drink only plain water when thirsty, avoiding juice or any sweet or salty beverages, and discontinue DI treatment regularly within 6 months after surgery,” “it is recommended to manage hyponatremia caused by SIADH with fluid restriction, while cerebral salt wasting syndrome causing hyponatremia requires treatment with hypertonic fluids mediated by brain natriuretic peptide,” “it is conducive to providing postoperative complication prevention health education to patients and their family members,” “it is recommended to conduct lifelong follow‐up and review for Cushing's disease (CD) patients after pituitary surgery, starting from 1 week postoperatively, with annual evaluation for those in remission,” “we recommend multiple measurements of serum sodium within the first 5–10 days after pituitary surgery; evaluating free thyroxine (T4) and prolactin within 1–2 weeks postoperatively are recommended to assess for significant pituitary insufficiency,” “it is advisable to establish a postoperative multidisciplinary follow‐up management team for patients undergoing pituitary adenoma surgery,” and “for patients with pituitary insufficiency, it is initially recommended to undergo endocrine evaluation every 6 months and receive hormone replacement therapy. Subsequently, follow‐up should be conducted every 1–2 years throughout the patient's lifetime. Long‐term follow‐up is recommended.” The impact of dietary interventions on DI is of low quality and needs to be interpreted with caution. The details of the evidence are presented in Table 4.

**Table 4:** Summary of best‐evidence recommendations for management of endocrine complications after pituitary adenoma surgery.

Evidence item	Content of evidence	Evidence level	Recommendation level
Endocrine evaluation	1. Monitor newly developed hormone level abnormalities, recommend comprehensive evaluation of pituitary, thyroid, gonadal, growth hormone axis and prolactin levels starting from 6–12 weeks postoperatively, periodically monitor pituitary function^20^, ^23^, ^26^	Level 2	B
	2. Patients diagnosed with preoperative pituitary insufficiency should be re‐evaluated postoperatively to confirm the need for hormone replacement therapy^26^	Level 2	B
	3. Morning serum cortisol should be monitored (1–5 days postoperatively)^23^, ^26^	Level 4	B
	4. If morning serum cortisol levels are too low at 6–12 weeks postoperatively, dynamic testing should be performed to evaluate pituitary–adrenal axis function^23^	Level 5	B
	5. Pituitary adenoma patients with normal HPA axis function should not use glucocorticoids postoperatively, which does not affect AI, surgical complications, and DI, DSH incidence^27^, ^28^	Level 1	A
Management of secondary adrenal insufficiency	6. Hydrocortisone is the preferred glucocorticoid replacement therapy for secondary adrenal cortical insufficiency^26^	Level 1	B
	7. It is recommended that patients with secondary adrenal insufficiency receive glucocorticoid replacement therapy after surgery and receive discharge health education on adrenal insufficiency precautions^19^, ^22^	Level 1	A
Management of water metabolism disorder	8. To screen for DI and SIADH, measure urine volume and fluid intake, measure urine specific gravity daily, and measure serum sodium every 6–12 h until 7–10 days postoperatively^19^, ^21^, ^26^	Level 1	A
	9. Use vasopressin as needed to treat DI and reassess efficacy, and avoid the use of ADH during SIADH episodes^20^, ^26^	Level 3	B
	10. It is not recommended to use DDAVP in the first week after surgery due to the risk of transient DI regression and the risk of SIADH occurring 7–10 days postoperatively^20^	Level 2	B
	11. It is recommended not to overtreat early postoperative DI to reduce the risk of hyponatremia during the antidiuretic phase^26^	Level 3	B
	12. Patients are advised to drink only plain water when thirsty, avoid juice or any sweet or salty beverages, and discontinue DI treatment regularly within 6 months after surgery^21^, ^23^, ^29^	Level 1	A
	13. It is recommended to avoid high‐salt foods such as pickles, processed foods, or adding extra salt, salad, and yoghurt to the diet^29^	Level 1	B
	14. It is recommended not to consume protein‐rich foods (eggs, dried fruits, cheese, chicken, legumes, and peas)^29^	Level 1	B
	15. It is recommended to avoid caffeine‐containing beverages that may cause diuresis, such as tea and coffee^29^	Level 1	B
	16. Mild hyponatremia can be managed on an outpatient basis by restricting fluid therapy, while severe hyponatremia requires hospitalization and may require short‐term use of hypertonic saline or ADH receptor antagonists, with caution to avoid overcorrection^26^	Level 4	B
	17. It is recommended to manage hyponatremia caused by SIADH with fluid restriction, while cerebral salt wasting syndrome causing hyponatremia requires treatment with hypertonic fluids mediated by brain natriuretic peptide^26^	Level 1	A
	18. It is recommended to restrict water intake after surgery to reduce the occurrence of delayed hyponatremia, but there is no unified consensus on the amount of water restriction, timing, and duration of water restriction^25^	Level 3	B
	19. It is recommended to increase intake during hot seasons and increase physical exercise^21^	Level 3	B
	20. It is recommended to provide postoperative complication prevention health education to patients and their family members^21^	Level 3	A
Management of special populations	21. Patients with Cushing's disease should receive GC replacement therapy based on cortisol levels after the first surgery^26^	Level 3	B
	22. It is recommended to conduct lifelong follow‐up and review for CD patients after pituitary surgery (starting from 1 week postoperatively, with annual evaluation for those in remission)^23^, ^26^	Level 2	A
	23. It is recommended to provide education on the disease, treatment plan, and expected outcomes after remission to CD patients and their family/caregivers^22^, ^26^	Level 5	B
	24. We recommend multiple measurements of serum sodium within the first 5–10 days after pituitary surgery; recommend evaluating free T4 and prolactin within 1–2 weeks postoperatively to assess for significant pituitary insufficiency^20^, ^22^	Level 1	A
	25. Serum GH and IGF‐1 in acromegaly patients should be measured 12 weeks or later after surgery^22^, ^23^, ^26^	Level 2	B
Follow‐up management	26. It is recommended to establish a postoperative multidisciplinary follow‐up management team for patients undergoing pituitary adenoma surgery^26^	Level 4	A
	27. It is recommended that patients with normal pituitary function undergo detailed biochemical diagnosis 1 year after surgery. If the results are normal, further endocrine follow‐up care should be discontinued^19^	Level 4	B
	28. For patients with pituitary insufficiency, it is initially recommended to undergo endocrine evaluation every 6 months and receive hormone replacement therapy. Subsequently, follow‐up visits should be conducted every 1–2 years throughout the patient's lifetime. Long‐term follow‐up is recommended^19^, ^23^, ^24^	Level 4	A
	29. It is recommended to perform the first MRI examination 3–4 months after surgery, followed by subsequent examinations every 3–6 months based on hormone levels and the patient's condition. Once the cure criteria are met, an annual MRI examination is recommended^24^	Level 5	B
	30. Detailed health education should be provided to patients upon discharge^24^	Level 5	B

Abbreviations: ADH, antidiuretic hormone; AI, adrenal insufficiency; CD, Cushing disease; DDAVP, desmopressin; DI, diabetes insipidus; DSH, delayed symptomatic hyponatremia; GC, glucocorticoid; GH, growth hormone; HPA, hypothalamic–pituitary–adrenal; IGF‐1, insulin‐like growth factor‐1; MRI, magnetic resonance imaging; SIADH, syndrome of inappropriate antidiuretic hormone.

## 4 DISCUSSION

This study summarized the best evidence for managing postoperative endocrine complications in patients with pituitary adenomas, including content in five aspects: endocrine assessment, management of AI, management of water metabolism disorders, management of special populations, and follow‐up management. It is recommended that healthcare providers fully consider the current physical condition, psychological status, clinical reality of patients with pituitary tumors, and patient preferences when applying evidence and choose evidence selectively and appropriately.

### 4.1 Prompt endocrine assessment

Evidence item 2 states that “Patients diagnosed with pituitary insufficiency before surgery should be re‐evaluated after surgery to determine whether hormone replacement therapy is necessary” because a significant proportion of patients may experience improvement in their endocrine function after adenoma removal. Jahangiri et al.^29^ reported that after pituitary adenoma surgery, 36% of patients experienced normalization of the thyroid axis, 18% of male patients experienced normalization of the gonadal axis, 41% of female patients experienced normalization of the gonadal axis, 29% experienced normalization of cortisol, and 22% experienced normalization of GH. However, postoperative recovery from pituitary tumors may require a longer period, and the recovery rate after 1 year is greater than the recovery rate after 3 months. Patients with acromegaly, compared to those with nonfunctioning pituitary adenomas, are more likely to recover adrenal function after surgery. The timing of re‐evaluation should be determined based on clinical judgment.^28^

Evidence item 3 suggests that “Morning serum cortisol levels should be monitored after surgery (1–5 days postoperatively).” American Endocrine Society has reported a strong correlation between morning cortisol levels <4–5 μg/dL and the occurrence of AI, while patients with levels >10–15 μg/dL have a lower likelihood of developing AI. If AI occurs, hormone replacement therapy should be administered, and adrenal function should be reassessed using a corticotropin stimulation test 6 weeks after surgery.^30^

Evidence item 5 states that “In patients with normal HPA axis function after pituitary adenoma surgery, it is recommended not to use glucocorticoids postoperatively, as it does not affect the occurrence rates of AI, surgical complications, DI, or DSH.” A high‐quality RCT compared the effects of glucocorticoid replacement therapy with no glucocorticoid replacement therapy on the recurrence rate of AI in patients with an intact HPA axis who underwent pituitary adenoma surgery. The study reported an AI occurrence rate of 11.0% in the group receiving glucocorticoid replacement therapy and 6.4% in the group not receiving hydrocortisone replacement therapy, with a difference of 4.6%. It is safe to not give glucocorticoids around the time of surgery to patients with normal adrenal function, with a 10% margin for safety.^25^ However, when AI occurs after surgery, prompt glucocorticoid replacement should be administered, and postoperative hyponatremia should be closely monitored. Excessive use of glucocorticoids may interfere with postoperative examinations and lead to unnecessary treatment at discharge. Nevertheless, the optimal cortisol threshold for diagnosing AI remains unclear, and future studies are needed to determine the safety of lower postoperative cortisol cutoff values and minimize the inappropriate use of glucocorticoids. Additionally, studies have shown that glucocorticoids may affect blood glucose homeostasis and electrolyte levels.^31^ More studies are needed to determine the impact of glucocorticoid use on surgical outcomes in pituitary adenoma patients with concomitant diabetes mellitus.

### 4.2 Evaluation and management of secondary AI

For patients who develop secondary AI after surgery, evidence item 6 suggests that “hydrocortisone is the preferred glucocorticoid replacement therapy for secondary adrenal cortex insufficiency.” The best practice guidelines recommend administering all patients intravenous hydrocortisone (e.g., 50 mg every 6 h) within the first 24 h following pituitary surgery, with rapid tapering to standard oral replacement doses (15–20 mg/day) over the next 48 h if the surgery is uncomplicated. Many experts currently support postoperative oral glucocorticoid administration rather than intravenous injection, provided that patients can tolerate oral intake.^32^ After discontinuing glucocorticoid, evidence item 7 suggests that morning plasma cortisol levels should be checked on postoperative Day 3, along with continued clinical assessment to determine the need for long‐term glucocorticoid replacement.^33^ Patients with secondary AI after surgery are advised on glucocorticoid replacement therapy and AI precautions at discharge. The typical daily dose of hydrocortisone at discharge is usually 15–30 mg/day (2/3 in the morning, 1/3 at noon; otherwise, the dose can be divided into three equal parts/day). Patients were advised not to adjust their dosage without further evaluation. During hospitalization, patients should receive training on dose adjustments for hydrocortisone dependency and keep an emergency card with an injection at the bedside.^17^

### 4.3 Management of water metabolism disorders

Water metabolism disorders may be related to intraoperative damage to the hypothalamus, pituitary stalk, or posterior pituitary lobe, which alters water metabolism controlled by ADH.^34^ These water metabolism disorders may occur due to a decreased release of ADH, leading to central DI, excessive release of ADH, water retention, and SIADH. Sometimes after pituitary surgery, a rare condition called cerebral salt wasting syndrome may occur. This condition is not related to ADH. To screen for DI and SIADH, urine output and fluid intake should be measured, urine specific gravity should be measured daily, and serum sodium should be measured every 6–12 h until 7–10 days postsurgery because central DI, characterized by a lack of ADH, typically occurs on the first day after surgery. SIADH, characterized by increased ADH release, usually occurs several days after surgery. In rare cases, the stages of central DI and SIADH may alternate, which can hinder proper diagnosis and treatment.^35^ Evidence item 9 suggests that “Use vasopressin as needed to treat DI and reassess efficacy, and avoid the use of ADH during SIADH episodes.” Short‐acting vasopressin is recommended because of its shorter duration of action, and desmopressin is not recommended for preventing temporary DI from converting to SIADH. It has shown that postoperative SIADH appears to be more common in patients with cardiac, renal, or thyroid disease, older age, lower blood pressure, lower body mass index, and who undergo postoperative lumbar drainage.^36^ Since the course of SIADH is usually transient, overtreatment of DI should be avoided in the early postoperative period to reduce the risk of hyponatremia when SIADH occurs in the second stage. Evidence items 12–15 present dietary plans for preventing DI based on the pathophysiology of ensuring adequate free water intake and a low metabolic load and plans for avoiding diuretic food intake to reduce solute load and minimize the occurrence of DI.^37^ Research has shown that nurse‐led dietary plans can attenuate the trend of DI, decrease the incidence of polyuria and hypernatremia, reduce vasopressin requirements, and shorten hospitalization time in patients undergoing pituitary adenoma surgery.^26^ Evidence item 18 demonstrates that restricting water intake has been shown to reduce the occurrence of delayed hyponatremia after surgery. However, different studies have different limits on daily water intake, ranging from 1 L to 2.5 L/day. Additionally, the duration of water restriction also varies, with some starting on the first day after surgery and others delaying until the fourth day after surgery. The duration of water restriction ranges from 4 days to 2 weeks postoperatively.^7^, ^13^ In the future, multicenter prospective studies should be conducted to fully evaluate the effectiveness of water restriction in patients after pituitary adenoma surgery and establish standardized water restriction protocols.

### 4.4 Management of special populations

Evidence items 21–24 guide the management of patients with CD after pituitary surgery. These patients were closely monitored for serum cortisol levels every 6 h starting 12 h after surgery. If the serum cortisol concentration drops below 138 nmol/L (5.0 µg/dL) or if the patient experiences symptoms of AI, glucocorticoid replacement therapy should be initiated, and lifelong follow‐up is recommended.^21^, ^24^ Since 5%–10% of CD patients may develop hyponatremia after surgery, which typically occurs between postoperative Days 5 and 10,^38^ the serum sodium concentration should be measured during this period. Thyroid function may also be affected by this procedure, as the half‐life of T4 is 5–7 days, leading to a potential diagnosis of hypothyroidism within the first week after surgery. Therefore, free T4 and prolactin levels should be evaluated within 1–2 weeks after surgery to assess for possible pituitary dysfunction.^39^ For patients with acromegaly, serum GH and serum insulin‐like growth factor‐1 (IGF‐1) levels should be measured at least 12 weeks after surgery. Postsurgical diuresis may occur due to a decrease in GH and IGF‐1 levels, and even in patients with complete resolution of acromegaly, it may take several months for IGF‐1 levels to return to normal.^40^ Therefore, serum GH and IGF‐1 levels should be measured at least 12 weeks after surgery.^41^ Currently, the best test for evaluating the resolution of GH or IGF‐1 abnormalities after pituitary surgery is the 75 g oral glucose tolerance test, which should reveal a GH concentration <0.4 ng/mL within 2 h after administration.^42^

### 4.5 Follow‐up management

Evidence item 26 states that “It is recommended to establish a postoperative multidisciplinary follow‐up management team for patients with pituitary adenoma surgery, including neurosurgery, endocrinology, radiology, and intensive care unit (ICU) healthcare professionals. In special cases, other disciplines such as gynecology, psychiatry, and mental health centers may also be included.” Evidence items 27–29 provide recommendations for follow‐up time for different patients. For patients with normal pituitary function after surgery, a detailed biochemical diagnosis is suggested for 1 year, and if the results are normal, further endocrine follow‐up care should be stopped.^19^ For patients with pituitary insufficiency, an initial endocrine evaluation should be conducted every 6 months, and hormone replacement therapy should be given. Subsequently, follow‐up should be conducted every 1–2 years throughout the patient's lifetime, and lifelong follow‐up is recommended.^17^, ^21^, ^22^ In addition, detailed health education should be provided to patients before discharge, emphasizing the importance of long‐term follow‐up in disease management and improving quality of life. A follow‐up card should be provided, informing the patient of the follow‐up process. Patients will receive an annual follow‐up questionnaire survey, and if there is an address change, the survey should be promptly communicated to the follow‐up physician and nurse.^22^

### 4.6 Limitations

This study has several limitations. First, the inclusion of literature from different countries and ethnic groups, taking into account racial and population variances, suggests that in clinical practice, a tailored approach should be adopted to select or enhance evidence based on the medical environment, surgical plans, and patient conditions for devising a rational management strategy for pituitary tumor complications. However, high‐quality intervention studies on postoperative endocrine complications of pituitary adenomas have clear research ideas, rigorous designs, and are valuable for reference. Second, given that there is only one original study on dietary plans included in this research without long‐term follow‐up, further investigation is warranted to evaluate the effectiveness of dietary interventions. Finally, as this study focused on consolidating the highest quality evidence available, it exclusively incorporated RCTs while excluding other types of original studies. With the increase in original studies, it will be possible in the future to conduct evidence synthesis and systematic reviews specifically on high‐quality original literature, further supplementing the content of evidence.

## 5 CONCLUSIONS

This study focuses on the management and follow‐up strategies for postoperative complications such as AI, temporary or permanent DI, and hyponatremia, providing valuable insights into the management of endocrine complications after surgery. However, due to a lack of long‐term follow‐up evidence, further studies are needed regarding the impact of dietary interventions on DI. Additionally, due to variations in practices among different hospitals, centers, and surgeons, as well as the potential differences in hormone replacement strategies for different types of pituitary adenomas, it may be necessary to conduct multicenter prospective studies and subgroup analyses to personalize medication for different patient types based on local circumstances. Furthermore, an exploration of suitable water restriction protocols for the Chinese population is needed to determine the optimal timing and duration of water restriction to reduce the occurrence of delayed hyponatremia.

## AUTHOR CONTRIBUTIONS

Zhongmin Fu and Shunwu Xiao were involved in the conception and design, or analysis and interpretation of the data. Xiaoju Miao drafted the paper. Xian Luo and Jun Wang critically revised the knowledge content. Zhangzhu Ren and Yuanyuan Wang conducted the literature screening and evaluation, while Guoping Mei and Zhongmin Fu performed the evidence synthesis. All authors agree to take responsibility for all aspects of their work.

## CONFLICT OF INTEREST STATEMENT

The authors declare no conflict of interest.

## ETHICS STATEMENT

This study integrated previous literature for evidence‐based analysis and was registered at the Evidence‐Based Medicine Center of Fudan University (http://ebn.nursing.fudan.edu.cn/, registration number ES20233124).

## Data Availability

The data that support the outcomes of this paper are available from the corresponding Author, Shunwu Xiao, upon reasonable request.
